# Professionals’ ideals of care in the context of technological innovation in healthcare, a focus group study

**DOI:** 10.1186/s12912-025-03908-x

**Published:** 2025-10-22

**Authors:** Ellen Ramvi, Birgitta Haga Gripsrud

**Affiliations:** Department of Caring and Ethics, Faculty of Health Sciences, University of Stavanger, P.O. Box 8600, Stavanger, 4036 Norway

**Keywords:** Ethics of care; healthcare personnel, Technology, Ideals of care, Conditions for care

## Abstract

**Background:**

Societally, we are witnessing unprecedented rapid advancements in medical and welfare technology, and technologies are increasingly embedded across healthcare services. Yet, little is known about how such developments might impact health professionals’ ideals of care. This study aimed to generate experience- and practice-near knowledge about healthcare professionals’ ideals of care, and to explore whether their ideals were affected by technological innovation in public healthcare organizations.

**Method:**

A qualitative approach was used, based on six focus group interviews and reflexive thematic analysis. The study enrolled 26 healthcare professionals, comprising registered nurses, specialist nurses, and physicians, from six clinical settings.

**Results:**

After identifying the range and use of technology in the respective clinical contexts within the six focus groups, we generated three reflective themes from our inductive analysis: (i) Healthcare personnel feel they can be the professionals they want to be when they have a good relationship with their patients. (ii) The conditions for caring relations vary in the different clinical contexts. (iii) Changes affecting care are not driven by technological innovation.

**Conclusions:**

Despite increasing technological integration in healthcare, professionals consistently upheld relational ideals of care. They felt personally responsible for providing relational care, and experienced guilt and distress when unable to do so. Systemic pressures, such as time constraints, documentation demands, and individualized responsibility, threaten these ideals, underscoring the need for broader institutional, political, and societal support to sustain professional relational care.

## Background

In most European countries, the welfare state has been subject to large political reforms [1]. Technological innovation has emerged as part of these reforms and has been a key driver in healthcare, whether it involves new devices or novel digital solutions and software [2, 3]. To illustrate, medical technology offers a range of tools that are intended to enable healthcare professionals to provide patients with a better quality of life by performing early diagnosis, reducing complications, optimizing treatment, and reducing the length of hospitalization [4]. Digital health technologies are claimed to revolutionize the practice of healthcare [5]. New technologies carry a promise of innovation in healthcare practices, for example by improving efficiency and communication for healthcare professionals or by giving a sense of empowerment and convenience or increased accessibility to patients [6].

Added to this backdrop is another hope: the growing expectation from policy fields, that new and more technology – if implemented and applied appropriately – can contribute to solving future problems associated with increased patient needs and stretched human and economic resources in healthcare [7, p. 257]. At this juncture, it is therefore crucial to know more about how technology-related innovations and hopes correspond with the ideals of care among healthcare personnel tasked with carrying out care work in practice.

### Theoretical perspectives

Care is a theoretical and substantial thematic pivot point in this study. The concept is ambiguous, and there are many ways of understanding it. In this study, we understand care as conditional for life, in that “care, and being in need of care at various points of your life, is a condition of our existence” [8, p. 1] as human beings. Care reflects our susceptibility to vulnerability and dependency and therefore stands out as something deeply relational that we cannot live well without.

Theoretically, we view care from two complementary perspectives: the ethic of care [9–11] and an iteration of care theory developed in psycho-social studies [12]. In healthcare, an ethic of care emphasizes a holistic caring practice and the relationship between carer and care receiver, in which “presence, trust and respect” and knowledge about the person, as well as an awareness of power asymmetries, are at the center [13, p. 1128]. According to such an understanding, the caring professional is oriented toward attentiveness, responsiveness, responsibility, and competency. We add to this perspective an understanding of care as a psycho-social phenomenon, emphasizing the intersubjective dynamics of care, which interlink the individual with relational and societal contexts (see e.g. Gripsrud et al. [14]). Following a psycho-social understanding, in our study we are interested in the dialectic between healthcare personnel’s experience and the social conditions for care, which means “paying attention to the conflictual and dynamic nature of both psychological and social processes,” which may have “mutual effects on social action and change” within the healthcare context (www.psycho-societal.org). Care, in this sense, is a psychosocial practice because it links what goes on in the inner worlds of caring professionals with what is also going on in the outer worlds, globally and nationally in socio-cultural, economic, and political contexts, as well as locally in institutions and organizations [15].

Another conceptual pivot in this study is professional identity and identity formation. Healthcare personnel highlight that being personally involved or “using oneself” is a prerequisite to gain insight into complex relational situations with patients [16, 17]. Heggen [18] defines professional identity as “.a more or less conscious perception of ‘me’ as a professional, in terms of which characteristics, values and attitudes, which ethical guidelines or which skills or knowledge constitute me as a good professional” (18, p. 324, our translation from Norwegian). We therefore view professional identity formation as something beyond the acquisition of work-related skills and knowledge: It relates to a process of becoming, an integrative development throughout one’s professional career, whereby the understanding of oneself as a professional and one’s core values and moral compass gradually take shape and change through situated experience. Through exercise of the professional role, conceptions of “myself” are produced and reproduced. Professional identity formation, as we wish to approach this, will be closely linked to self-understanding; “myself as a professional person.” Thus, the focus in this study is on healthcare personnel’s experience of ideals of care and professional identity, and we ask if they can be the professionals they want to be in a healthcare context characterized by technological innovation.

## Method

### Aim and research questions

This study was designed to gain experience- and practice-near knowledge about healthcare professionals’ ideals of care and whether their ideals are affected by technological innovation in public healthcare organizations. Our research questions were as follows: How do professionals in public healthcare describe their current ideals of care? Do they experience that they can be the professionals they want to be?

### Research design

The study’s qualitative design facilitated in-depth investigation of our research topic, allowing us to capture complexity, interconnections, and nuances of experiences and meanings. It was based on six focus group interviews with healthcare personnel whose clinical practices involved using different types of technology to different degrees. We approached the transcribed focus group interviews with reflexive thematic analysis.

### Participants and research context

To cover variations in the types of technology used, and professionals’ ideals and attitudes toward technology in healthcare, we recruited healthcare professionals from municipal services (community health nurses in school health services and a clinic for youth, community health nurses working in a health center for children aged 0–5 years, as well as nurses working in home care), and specialist health services (nurses and physicians in a neonatal intensive care unit and a department of respiratory medicine, and nurses in a dialysis unit). Descriptive characteristics of the participants are presented in Table 1.

To recruit the participants, we sent an information letter to the managers of selected units/wards. We asked for 3–6 voluntary participants. The managers who accepted the invitation to take part in the study helped us with the recruitment process in their department.

**Table 1:** Participant characteristics

Number of years of experience at the unit	Education		
	Registered nurse	Registered nurse with further education*	Physician
**0–2 years**	1	2	
**3–5 years**	6	1	1
**6–10 years**	2	3	1
**10 or more years**	2	6	1
**In all**	**11**	**12**	**3**

*The registered nurses with further education specialized in neonatal care (n 3), public health (n 7), or both public health and psychiatry (n 2)

Altogether 26 health personnel, all female, agreed to take part in the study. Their work experience was varied, but as can be seen from Table 1, only one of the nurses had worked in the unit for less than three years. Notably, 12 of the 23 registered nurses had further specialized education. The participant sample was, in other words, constituted by experienced and well-educated health personnel.

### Data collection

We chose to collect data through focus groups because we wanted to promote discussions of everyday practice and concerns that are consistent with the ethic of care. Focus groups are increasingly used in critical and interpretive inquiry [19] and have been applied across a broad spectrum of onto-epistemological knowledge positions. A crucial strength of focus groups is that they can be conducted as “dialogic events within which power relations between researchers and research participants are diminished and people collectively interrogate the conditions of their lives to promote transformation” [19, p. 694].

We conducted six focus group interviews: three with healthcare personnel in primary care services (n 13) and three with healthcare personnel from the specialist health services (n 13).

With relatively small focus groups of three to six participants, we sought to increase the depth of discussion. In the role of moderators, we – the researchers (ER and BHG) – tried to facilitate the conversation among participants while not imposing questions and comments. We used an interview guide with only a few questions to spark a conversation among the participants. Any further follow-up questions were aimed at increasing the depth of discussion. One advantage was that the participants were from the same unit and thus used to talking to each other, and we did not need to give them much time to introduce themselves. The focus groups lasted approximately 1 h and took place in a quiet location at the respective department/unit. The interview guide was the same for all six focus groups, with questions only slightly adapted to target the specific unit’s context of care:

1. What kind of technology do you use within your unit? (*to set the “frame” for subsequent questions*)
2. Has the attitude to care and ideals of good care (for this specific group of patients) changed in the culture in this unit? (*this included the question of what is the ideal for care at the unit*)
3. Can you be the professional you want to be in this unit? (*to capture key aspects of the self-understanding of the professionals*)

The focus groups were recorded and subsequently transcribed verbatim for analysis.

### Data analysis

We opted to approach the transcribed focus group material with reflexive thematic analysis [20, 21], which embraces “the values of a qualitative paradigm, centering researcher subjectivity, organic and recursive coding processes, and the importance of deep reflection on, and engagement with, data” [20, p. 593]. To make the approach more intelligible, Braun and Clarke [22] explicate a six-phase process for data engagement, coding, and theme development, involving: (1) data familiarization and writing familiarization notes; (2) systematic data coding; (3) generating initial themes from coded and collated data; (4) developing and reviewing themes; (5) refining, defining, and naming themes; and (6) writing the report. However, they emphasize that in practice, these phases do not necessarily take place sequentially. Correspondingly, our experience was that these six phases oscillated or at times blended together, as the analysis becomes increasingly recursive. As researchers interested in psycho-social dynamics between inner and outer worlds, we value the knowledge impact of reflexivity, where themes come about inductively, deductively, *and* generatively through active engagement with the data over time.

### Ethical considerations

The study was approved by the Norwegian Social Science Data Service, Bergen, Norway, project number (556960), and followed the Declaration of Helsinki [23]. Researchers provided participants with a verbal description of the study and a written information sheet. Participants gave written consent to participate in the study and were informed about their right to full confidentiality and their right to withdraw from the study at any time. Participant and third-party anonymity are preserved in the text. The international standard for authors [24] was followed.

## Results

### The range and use of technology

As mentioned above, we wanted to set the initial frame for the subsequent questions by asking the focus groups what kind of technology they used within the work unit. In all six focus groups, enrolled from either municipal or specialist health services, information and communication and computer technologies (laptops, tablets, mobile phones, e-portals, webpages, social media, journals, and referral systems) appeared by far the most prevalent types. For example, electronic patient journal systems were in daily use for documentation, referrals, prescriptions, etc. for all the groups.

Beyond the standard and specialized communication and information technologies, the focus groups referred to the use of both standard medical technologies (for example, tools for the measurement of temperature or oxygen saturation, and blood pressure monitors), as well as more specialized medical technologies (audiometry for assessing hearing, respirators or BiPAP ventilators (bilevel positive airway pressure) as breathing support, standard dialysis machines, and home dialysis machines) relating to their specific clinical fields. Both the focus groups from neonatal and dialysis units rely on vital and lifesaving equipment every day. The focus group from homecare services referred to more numerous technologies than any other group: medicine dispensers, telemedicine technology, safety alarms, fall alarms, sensors, and GPS tracking, as well as medical technology/equipment (catheters, tubes for draining or feeding, pain pumps, home dialysis).

The focus group from the health center for children aged 0–5 years laughed as they said “we’re pretty low-tech” but indicated that they appreciated new technology that could help with administrative duties. Surprisingly to us, the focus group from respiratory medicine also said that they did not think of themselves as representative of a high-tech environment.

Having provided this brief overview of new and established technologies used in the clinical contexts of our focus groups, we continue with the three themes we generated through the data analysis: (i) Healthcare personnel feel they can be the professionals they want to be when they have a good relationship with their patients. (ii) The conditions for caring relations vary in the different clinical contexts. (iii) Changes affecting care are not driven by technological innovation.

### Theme 1: Healthcare personnel feel they can be the professionals they want to be when they have a good relationship with their patients

Despite working in different sectors and specializations with different uses of technology, our participants shared the same ideal for care: that a good relationship with patients and their families was the ideal. When they tried to specify what a good relationship was in their view, they said it was to have continuity in the relationship, being able to follow up patients and to be with them during difficult moments, and forming emotional bonds by talking with, listening to, and ‘seeing’ the patient. Furthermore, in a good relationship, they want to consider the patient’s perspective and show respect for individual differences and needs. In a good relationship, they wanted to be able to sometimes go beyond what is strictly necessary.

Failure to provide this ideal care – the care they wanted to give – entailed emotional challenges, such as feelings of guilt, falling short, and discouragement. One of the participants said she often went home feeling guilty “for not doing as good a job as I should” (community health nurse in the focus group from school health services and clinic for youth), and this experience was echoed in the other focus groups.

Although technology was more or less part of care in all the work units of the participants, a striking finding was how little the participants talked about technology related to the ideal of care and how little importance technology seemed to have for their experience of being the professional they want to be. As researchers, we were surprised by this, as we had initially expected to find a greater influence of technology on the professionals’ ideals in clinical contexts that are more technology oriented. We had also expected technology to come much more into conflict with professionals’ ideals. But this was not the case. A repeated reflection from our participants was that technical equipment should, to the least extent possible, come in the way of contact with the user or patient or next of kin. To be the professionals they want to be, the participants wanted to feel contact in their relationships, to use their professional judgment, and to experience meaning in their work.

### Theme 2: The conditions for caring relations vary in the different clinical contexts

There were remarkable differences among the focus groups when it came to the cultures of care within their work units, and the conditions for care varied between the groups. Every focus group mentioned that enough time and staffing were what they needed to provide good care and to experience contact with the patient or user.

Those groups who felt they had a positive care culture emphasized that mistakes were tolerated and could be learned from in their workplace. They also felt that everyone helped each other as colleagues and worked together as a team.

Those focus groups who felt they had a poor culture of care in their unit said that their workplace had a managerial, finance-driven orientation and an absence of value-driven leadership. In one of the focus groups from the hospital units, for example, they said that what was best for the patient was what was most expensive, and that’s why “the quality of care will never be very good because [in our unit] it is always about finances.” Another replied: “Sometimes I feel like a rat in a hamster wheel; it’s really kind of like you work in a factory.” These sentiments were more or less pronounced in the other focus groups.

A lack of routines for processing emotional experiences at work was also said to be common in the units with poor care cultures. In one of the focus groups, from an organizational culture with no routines for reflecting over or processing emotions, one of the participants said that in the event of a very serious situation in the unit, “We close the door to the nurses’ station and blow out. I think everyone has shed tears more than once in this department. It’s kind of like – now I’m – now I’m starting to cry – can somebody just hold me!”

The burdensome experience of feeling alone with the responsibility for patients was a finding more common in the focus groups from municipal health care.

### Theme 3: Changes affecting care are not driven by technological innovation

All six focus groups pointed to one change as being of concern, namely an increased obligation to produce documentation. Some of the focus groups, for example the one in home care services, felt that there was increased standardization of their work and that guidelines were decided and developed without involvement from those who perform the service. Participants claimed that this increased standardization and documentation shifted the focus away from the patient and possibly hindered the caring relationship. As mentioned, all the participants believed that being able to use their professional judgment was important. The work should therefore not become routine or “assembly line-like” (nurse in the focus group from health center for children aged 0–5 years). In the focus group from the homecare service, they explained, “Care requires that you know the patient a little, so it’s difficult to have one template for everyone.”

The changes that were foregrounded by our participants with consequences for care were not technological innovations. The participants talked instead of an increased burden of, for example, mental health issues and complex psychosocial challenges such as domestic violence and substance use, which also indicated a turn toward more complexity and wider areas of responsibility, not only for the patient but also for the patient’s family. This required more interprofessional collaboration and a wider spectrum of knowledge to be able to give good care. Perhaps in light of this, in all the focus groups, participants talked about the need for developing more or new competence because demands for what they should be able to do and know as healthcare professionals were constantly increasing.

## Discussion

**Fig. 1:**
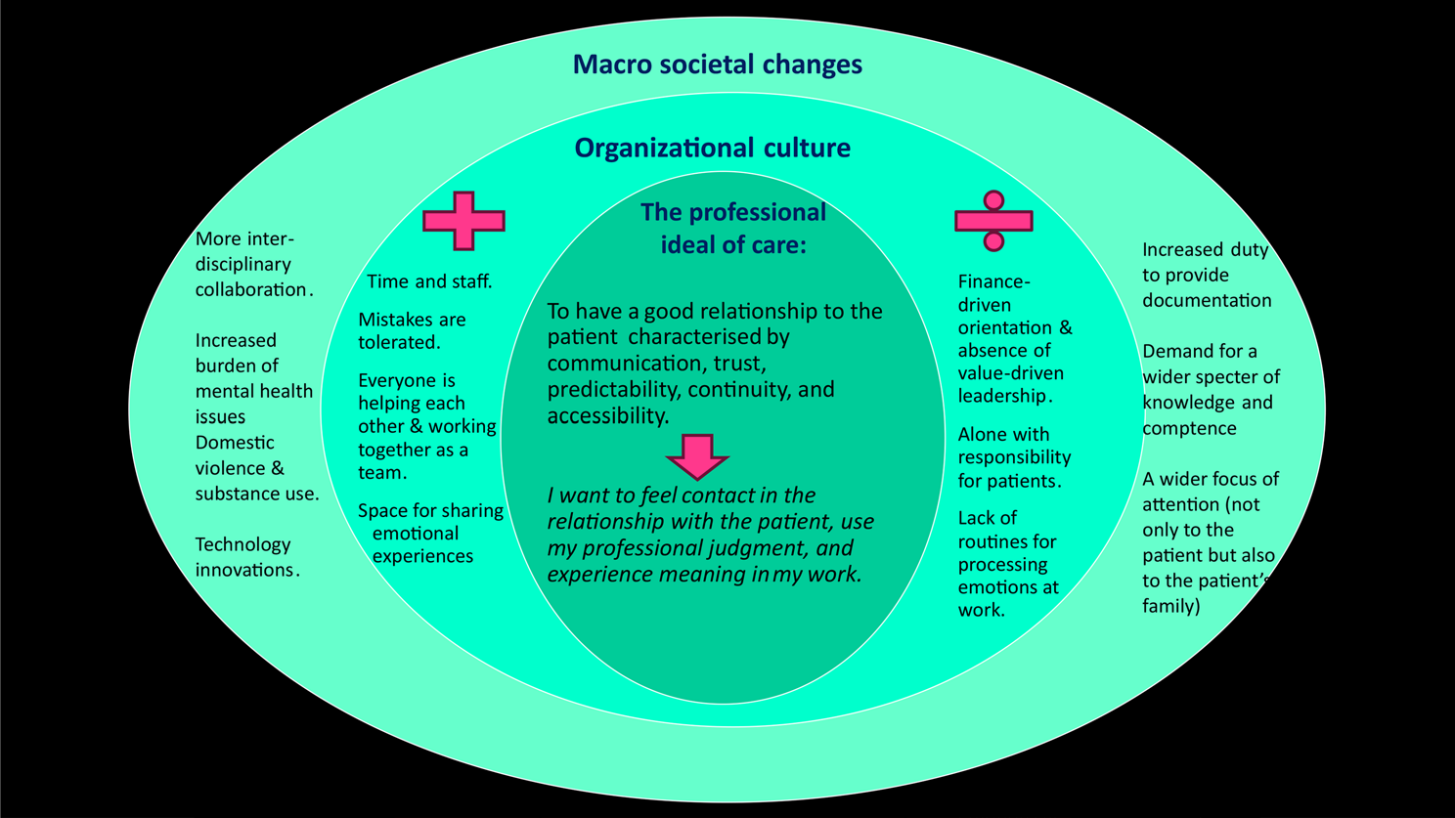
Professionals’ ideals of care in public healthcare

The purpose of the study was to gain new experience- and practice-near knowledge about healthcare professionals’ ideals of care and whether their ideals are affected by technological innovation in healthcare organizations. In Fig. 1, we present the results in a model to illustrate interlinking levels when it comes to a psycho-social understanding of professionals’ ideals of care in contemporary public healthcare. At the core is the individual – the professional ideal. The next circle illustrates the organizational culture, with what the participants said were positive or negative institutional factors for care. In the utmost circle, are their descriptions of wider societal changes with consequences for care. In our discussion, we pay attention to both psychological and social processes and attempt to link the individual professional ideal of care with what is going on in the outer world – in the institutions and in society.

The way the professionals describe their ideals of care is largely congruent with care ethics theory [9–11]. They emphasize care as situational and practice-near, showing how relationality still remains at the core of care practices. The ethic of care, expressed by our participants, emphasizes the importance of holistic care, in which healthcare personnel see the individual patient and their needs rather than having a standardized or one-size-fits-all approach. This resonates with Tomkins and Bristow’s [25, p. 132] claim that the ethic of care represents a powerful ethic of the particular.

However, the conditions for practicing the ethic of care were not the same for all participants. Some of the focus groups talked about increasing standardization of their work and guidelines for standardization being decided and developed without the involvement of those who will perform the service. In home care, for example, participants felt that they worked more and more following rules and routines – which frustrated the nurses. Cultures of “technical rationality” [26, 27] were a hindrance to providing professional holistic care. When professionals feel governed by rationalities or value systems that fail to foreground the ethic of care, they also feel that they fail to be the professional they want to be. The Nordic welfare states have traditionally supported “care-friendly” and “care work-friendly” contexts, and have even been referred to as “caring states” [8, pp. 2–3]. They have therefore been considered less likely to suffer the crises of care associated with New Public Management reforms, as seen for example in the UK. Nevertheless, the Nordic welfare states have also been “exposed to major challenges following the turn to neoliberal politics and the financial crisis” [8, p. 3]. We heard all participants mention that adequate time and staffing were a condition for being able to practice the ethic of care – and it was precisely time and staffing that were said to be in short supply to a greater and lesser extent in the various units. What resulted from not being the professional they want to be under such circumstances was feelings of guilt, troubled consciousness, and discouragement– all emotions that may affect job motivation and the ability to give good care.

Coming from a psycho-social perspective, we understand that our participants were in a difficult position. They appeared to have internalized demands from society about efficiency, and at the same time, they experienced irreducible demands from their professional and personal ideal to live up to the ethic of care, i.e., to be a person who cares. In addition, they experienced that the complexity of what they were expected to help patients with had become greater and thus entailed expanded requirements and competences.

Buvik et al. [28, p. 31] claim that working alone in the context of responsibility constitutes a possible tipping point where the emotional demands turn into a perceived or actual burden. The difficult feeling of facing immense demands may be one of the reasons our participants emphasized the importance of teamwork and peer support.

A professional’s self-image is linked to what kind of caring relationships they are able to have with their patients [29]. It can be difficult and shameful not to be able to live up to their ideal, and perhaps especially for women [30], who are conventionally expected to possess a “natural” ability to care. In line with previous research, our findings show that it is burdensome for the professional to feel that one is not enough and to feel responsibility for other people [28, 31]. Feelings of troubled conscience, powerlessness, and helplessness may, when interpreted from a psycho-social perspective, unconsciously lead healthcare personnel to avoid these feelings, and they may distance themselves from the person they are supposed to be helping in order to minimize the intensity of the emotional experience [32]. A concern is that this may happen to healthcare personnel who, over time and constantly, carry feelings of inadequacy. We can assume that the feeling healthcare personnel describe of troubled conscience will affect not only their professional self-esteem but also their self-estimation. It can be challenging for healthcare personnel to put into words the difficult feelings they get from not being who they want to be, and what problems and consequences this can have for the work they have to do. The intertwining of professional and personal identity can make it difficult for healthcare professionals to ask for the help they need with problems that can be experienced as both very personal and difficult to define and articulate. The gap between the ideal of care and the reality of conditions for care may challenge healthcare personnel’s perception of having value as a professional and may threaten their personal and professional identity.

The situation we outline here can perhaps best be described as an indication of moral distress. A recent scoping review [33] identified sources of moral distress in nursing, which align with our findings: Key challenges included lack of resources and autonomy, work overload, and institutional hierarchy, all of which negatively impact care quality. According to Peter and Liaschenko [34, p. 340], nurses feel moral distress most intensely when “they realize that they can never reach their ideals of patient care that are central to their moral identities as nurses.” Coming from a psycho-social understanding of care, we would argue that all healthcare professionals need to be in touch with their own responses, reactions, and emotions in order to establish and engage in caring relationships with patients and users. Such reflexivity informs their life-long learning as a professional becoming [35]. The ideals and realities of relational care are thus informed by professionals’ self-understanding and learning, in turn shaped by personal life and work experiences as well as the societal and institutional contexts which frame relational work in healthcare [36, 37].

### Limitations

Our findings are based on focus groups from three different municipal services as well as three different specialist health services. We wanted to enroll both high-tech and low-tech units to capture variations in care ideals. A potential limitation of our research design is that participants in the focus groups represent very different work environments. A narrower scope for recruitment could perhaps have helped deepen our analysis. For example, we could have focused on a specific technology or a specific healthcare specialization, which might have enabled a more thorough analysis of the ideals related to that particular care context. This was a choice we had to make when developing our design.

In retrospect, we see that the broader approach we opted for allowed us to identify striking similarities across the different focus groups and work units, both in terms of care ideals and in what they perceived as barriers to providing such care. What varied between the groups was the conditions for being able to deliver the kind of care they considered appropriate.

The participants were approached through the management of the respective units. We did not provide any selection criteria for recruitment but stated that we were interested in participation from both physicians and nurses where relevant or possible. The managers distributed our information letter during recruitment. However, we cannot know whether the managers made their own selections when inviting staff to participate in our focus group study.

The final sample did not include any male participants. Our goal was to achieve the greatest possible diversity in the sample, not to analyze different backgrounds (such as profession or gender) separately or in relation to each other. We therefore consider it a limitation of our material that it consists only of women, as male nurses might hold different experiences and care ideals. Similar limitations apply to ethnicity, as participants were, with one exception, professionals with a Norwegian ethnic background.

## Conclusions

In our study, we found that ideals of care among healthcare personnel seem to be more or less “timeless” and are largely unaffected by embedded technologies. Regardless of the different healthcare sectors and specializations our participants worked in, and their different uses of technology, they shared an ideal for care: a good relationship with the patient/user characterized by communication, trust, predictability, continuity, and accessibility. The experience of being in contact with the patient/user in the relationship, the ability to use their professional judgment, and experiencing meaning in the work were deemed important for the participants to feel like the professional they wanted to be. Lack of time and personnel was perceived as the most significant cause of inadequate care. Changes that affect care are more complex and wider areas of responsibility, together with an increased duty to provide documentation and follow standardized work guidelines. When it comes to technology, health care personnel’s main concern was that it should not come in the way of relational contact with the patient/user. Failure to provide the care they wanted to give entailed considerable emotional challenges. The individualization of responsibility for maintaining relational quality care may lead healthcare professionals to quit their jobs. Another potential consequence is that professionals may become less capable of caring, as they are likely to defend themselves psychologically against facing the guilt and moral distress associated with experiences of failures of care. These scenarios should be of grave concern for the welfare state and the technology developers, suppliers, public healthcare institutions and leaders within it. Therefore, the challenge of providing and sustaining professional relational care should be addressed more extensively at institutional, political, and societal levels.

## Data Availability

The datasets used in the current study are available from the corresponding author on reasonable request.
